# Feasibility, Acceptability, and Preliminary Performance of Check In for Exchange of Clinical and Key Information: A Communication Guide to Facilitate Pre-Encounter Huddles with Medical Interpreters Prior to Conversations Around Serious Illness

**DOI:** 10.1177/26892820251390817

**Published:** 2025-10-27

**Authors:** Mei-Ean Yeow, Daniel K. Partain, Heather J. Carmack, Megan Brandeland, Graciela D. Porraz Capetillo, Karen M. Fischer, Abraham Labrada Santiago, Ibrahim S. Karakus, Amelia Barwise

**Affiliations:** ^1^ Division of Community Internal Medicine, Geriatrics and Palliative Care, Mayo Clinic, Rochester, Minnesota, USA.; ^2^ Robert D. and Patricia E. Kern Center for the Science of Health Care Delivery, Mayo Clinic, Rochester, Minnesota, USA.; ^3^ Language Services, Mayo Clinic, Rochester, Minnesota, USA.; ^4^ QHS- Clinical Trials and Biostatistics, Mayo Clinic, Rochester, Minnesota, USA.; ^5^ Division of Pulmonary and Critical Care Medicine, Mayo Clinic, Rochester, Minnesota, USA.; ^6^ Biomedical Ethics Research Program, Mayo Clinic, Rochester, Minnesota, USA.

**Keywords:** conversations around serious illness, goals of care, medical interpreters, non-English Language preference

## Abstract

**Background:**

Patients with non-English Language Preference are at risk of adverse health outcomes, particularly at end of life and during serious illness. Medical interpreters often feel unprepared to interpret conversations about serious illness. Best practice guidelines recommend a pre-encounter huddle between clinician and interpreter to better prepare both providers. The CHECK-IN (Check in for Exchange of Clinical and Key Information) guide is a simple communication tool designed to facilitate this pre-encounter huddle. We describe the results of a pilot feasibility study of the CHECK-IN guide performed in a simulation environment. The primary objectives of the study were to evaluate the feasibility, acceptability, and preliminary performance of the CHECK-IN guide.

**Methods:**

This U.S.-based study is a single-center simulation-based nonblinded randomized pilot feasibility study. Participating clinicians were randomized to usual practice (control) vs. introduction to the CHECK-IN guide (intervention). Clinicians completed pre- and post-session surveys. Participating clinicians were evaluated using the Faculty Observation Rating Scale (FORS), Interpreter Scale (IS), and Interpreter Impact Rating Scale (IIRS).

**Results:**

Participants had a highly favorable opinion on the acceptability, appropriateness, and feasibility of the CHECK-IN tool; 91% of participants agreed/strongly agreed on the acceptability of the tool, 91% agreed/strongly agreed on the appropriateness of the tool, and 100% agreed/strongly agreed on the feasibility of the tool. There were no statistical differences between control and intervention groups for the IS, IIRS, and FORS scores.

**Conclusion:**

The CHECK-IN guide is a promising tool to guide a pre-encounter huddle between clinician and interpreter, thereby improving interpreter-mediated communication when having conversations about serious illness.

## Introduction 

There are currently over 25 million people in the United States with Non-English Language Preference, previously referred to as Limited English Proficiency.^1^ In health care settings, poor communication, and lack of awareness of the unique cultural and religious needs among this population can lead to adverse health outcomes across the spectrum of care, including at the end of life (EOL) and in patients with serious illness.^2–7^

Medical interpreters play an important role in the care of patients with non-English language preference. While a large body of work highlights the benefits of engaging professional medical interpreters to promote improved communication, clinical outcomes, and satisfaction with care among patients,^8–10^ a paucity of research has focused on preparing the clinician-interpreter dyad for conversations about serious illness. During serious illness conversations, interpreters can help bridge cultural as well as linguistic gaps in communication between patients and clinicians. Interpreters can also share insights with non-English language preference patients about the hospital health care culture and decision-making processes.^11^

While many clinicians may view the interpreter’s role solely as a language conduit, best practice guidelines from the International Medical Interpreters Association state that a medical interpreter’s role includes not only functioning as a linguistic conduit, but also acting as a cultural mediator and at times, patient advocate.^12^ Indeed, in a study by Silva et al.,^13^ interpreters felt that their role extended beyond language interpretation to include acting as cultural brokers or mediators, helping to contextualize difficult-to-explain terms.

Although medical interpreters are frequently engaged to assist with serious illness conversations, studies show that many interpreters feel unprepared and uncomfortable with these conversations.^13,14^ The goals of the clinical encounter are often not shared with the interpreter beforehand, and interpreters are often not included in team debriefing sessions.^15^ Additionally, interpreters acknowledge that these difficult encounters can evoke strong emotions, but they often lack the resources to help process these emotions.^16,17^ Many interpreters advocate for more training and support for both interpreters and clinicians in this area.

Prior research has established that interpreters should be integrated into the team for patients with non-English Language preference including during huddles, handoffs, briefings, and debriefings.^18,19^ A brief pre-encounter huddle between the clinician and interpreter just before meeting with the patient and family can be beneficial to establish a mutual understanding of the purpose of the encounter.^5,20^ The huddle may involve sharing essential clinical information about the diagnosis, prognosis, and treatment options, as well as leveraging the interpreter’s cultural knowledge and expertise.

Unfortunately, research shows that pre-encounter huddles with interpreters occur infrequently, with a recent survey of interpreters showing just over 70% never or seldom meet with the patient’s medical providers prior to a goals of care meeting, and only about 52% report they are usually or always treated as part of the medical team.^15^

No empirical research currently exists to guide health care teams or institutions in optimal approaches to implementing such a pre-encounter huddle. Our previous research looked at improving effective collaboration with medical interpreters and designing the CHECK-IN (Check in for Exchange of Clinical and Key Information) guide to facilitate a high-quality pre-encounter huddle.^21^ This guide provides a structured outline for the pre-encounter huddle with medical interpreters and encourages bi-directional sharing of key information prior to serious illness conversations. The guide prompts clinicians to share information including the purpose of the meeting, key clinical information to prepare the interpreter, and prompts interpreters to share important contextual cultural information to better prepare the clinician. This will allow the interpreter to be more emotionally and linguistically prepared for a potentially challenging and sensitive discussion and improve the clinician’s cultural sensitivity in leading the discussion, thereby improving the overall quality of the conversation. The objective of the current study was to build upon our previous research by establishing feasibility, acceptability, and preliminary performance of the guide among clinicians. We describe the results of a pilot feasibility study of the CHECK-IN guide conducted in a simulation-based environment.

## Methods

### Study design and setting

This study was a single-center simulated, nonblinded randomized pilot feasibility study. We held multiple simulation sessions from August 2024 to February 2025. Participants who agreed to participate in the study were randomized to either usual practice (control) or usual practice plus introduction to the CHECK-IN guide (intervention). The study was conducted at Mayo Clinic in Rochester, Minnesota, USA, a quaternary care academic medical center. The study sessions were held at our institution’s Simulation Center, which has a large, standardized patient program and supports multiple educational and research initiatives. The Mayo Clinic Institutional Review Board approved the study as exempt (ID-24-002923). Approval was granted by our institution’s Education Research Committee to include trainees in our study.

### Oral consent was obtained from each participant

#### Scenario design

The scenario was designed by study faculty (M.-E.Y., D.K.P., H.J.C., A.L.S., A.B.), with input from the simulation center personnel and the medical interpreter on the study team (G.D.P.C.). The scenario focused on a serious illness conversation, including discussing the transition to hospice care between a clinician, Spanish-speaking patient, and bilingual family member, and the encounter was facilitated by a medical interpreter. The clinician participant had to break bad news about cancer progression to the patient/family member and discuss the consideration of hospice care (see Supplementary Appendix A1 for case scenario).

#### Study participants

Inclusion criteria included any hospital-based clinician (physicians, advance practice providers, residents/fellows) who care for culturally and linguistically diverse patients with serious illnesses and had experience working with medical interpreters. Exclusion criteria included having specialty training in Hospice and Palliative Medicine and being fluent in Spanish.

We recruited participants through word of mouth, e-mail, paper flyers, presentations at division meetings (Hematology/Oncology, Pulmonary/Critical Care, Community Internal Medicine), and contact with Internal Medicine Chief Residents as well as Fellowship Directors (Hematology/Oncology, Pulmonary/Critical Care, Geriatrics, Gynecology-Oncology).

Participants received a small monetary compensation for their time in accordance with institutional regulations.

#### Intervention.

**FIG. 1.**
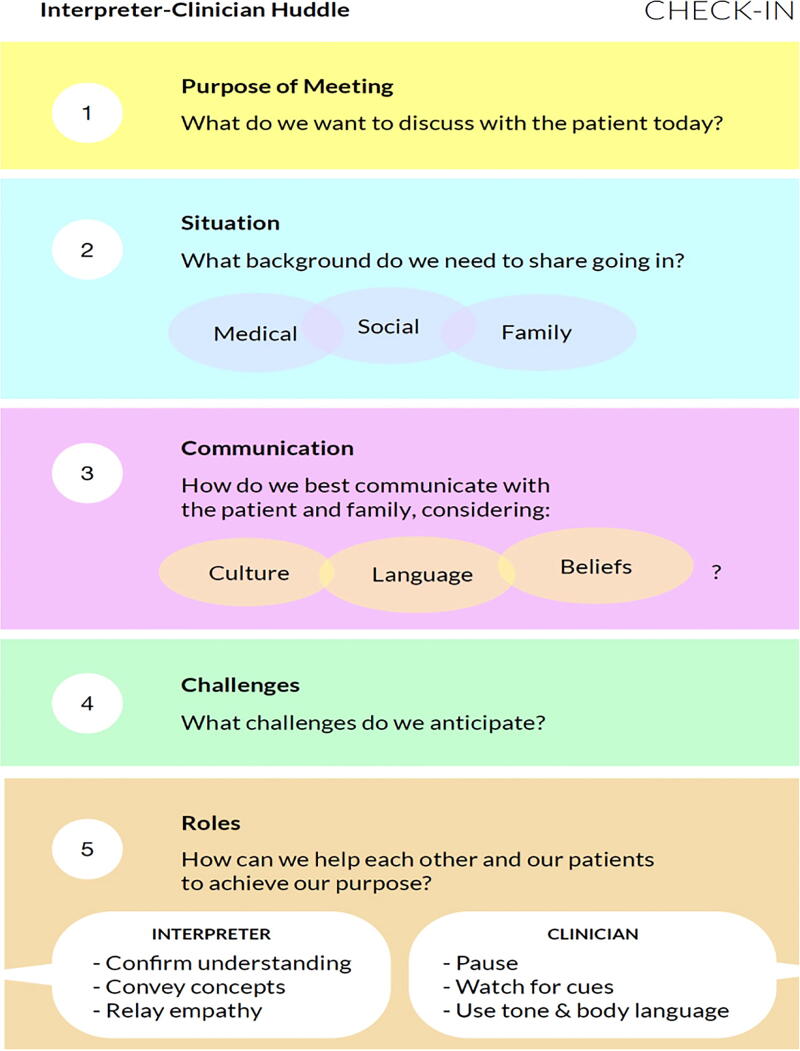
CHECK-IN guide. CHECK-IN, Check in for Exchange of Clinical and Key Information.

The CHECK-IN guide (see Fig. 1) is a simple bedside tool designed to facilitate collaboration during a serious illness conversation by prompting exchange of relevant sociocultural and clinical information between clinician and interpreter prior to the encounter with the patient and family.

#### Study procedures

For each study session, the participant completed a pre-encounter huddle with the medical interpreter followed by a simulated family meeting with the patient and family member. Both the pre-encounter huddle and family meeting were observed by two study faculty. The same medical interpreter (G.D.P.C.) acted as the interpreter in all the study sessions, and the patient and family member dyad were both portrayed by trained standardized patients (SPs)/actors.

#### Pre-encounter huddle

All participants engaged in a pre-encounter huddle. Those in the control group met with the interpreter and had an opportunity to ask questions. Those in the intervention group were given the CHECK-IN guide prior to meeting with the interpreter and were also given the opportunity to ask questions. In the pre-encounter huddle, if the clinician inquired about cultural or religious aspects of care, the medical interpreter shared relevant information with the clinician. The interpreter also shared with the participant that ‘hospicio’ was a false cognate in Spanish for “hospice” (*hospicio*—a home for indigent or unhoused persons that often carries a negative connotation for patients and families) and facilitated the use of more culturally sensitive terminology.

#### Patient encounter/family meeting

We included cultural considerations in the scenario and observed if the participant (1) elicited the patient’s preferences for information and approach to decision making, (2) asked about the role of culture and spirituality, (3) explained EOL care/hospice in a culturally sensitive manner, and (4) assessed understanding using ‘teach-back’ technique.

### Measures

#### Clinician participant surveys

Participants filled out both a pre- and post-session survey. Demographic information as well as information about training and clinical experience were collected in the pre-session survey. In the post-session survey, we asked participants to rate the perceived realism of the simulated family meeting.

Participants in the intervention group also assessed the feasibility/acceptability of the CHECK-IN guide using questions adapted from Weiner et al.’s Acceptability of Intervention Measure, Intervention Appropriateness Measure, and Feasibility of Intervention Measure.^22^

#### Faculty observations

Study faculty, the interpreter, and the actors completed evaluations of the pre-encounter and simulated family meeting encounter for each participant.

The outcome scales used were the Interpreter Scale (IS, completed by the interpreter), Interpreter Impact Rating Scale (IIRS, completed by the 2 actors), and the Faculty Observation Rating Scale (FORS, completed by 2 observing study faculty).

The IS is a 13-question validated instrument designed to measure communication skills as observed by trained interpreters, focusing on objective verbal and observable behaviors and minimizing interpreters’ emotional responses to questions. The IIRS is a seven-question validated instrument designed for use by SPs in any language encounter; it emphasizes patient-centeredness and observable behaviors as a patient with emotional engagement in the encounter. The FORS is a behavioral scale that was developed for faculty to assess interpreter-mediated communication skills among medical learners.

The IS, IIRS, and FORS are validated scales that have been validated in simulation encounters involving medical interpreters^23^ (see Supplementary Appendix A2).

We also designed a study observation checklist for the pre-encounter huddle and patient scenario to capture the cultural considerations of the encounters.

#### Data collection

All data (surveys and outcome measures) were completed on paper. Data were de-identified and entered and stored securely on RedCap.

### Statistical analysis

All statistical analyses were planned and performed in collaboration with a biostatistician (KF). Demographics for the two study groups (control and CHECK-IN Intervention) were described using frequencies and percentages. For the IIRS survey and FORS survey, both were scored by summing up the individual answers to get a composite score. Five participants that had one missing value in the FORS survey, which was filled in for the composite score by averaging the other answers. Cronbach’s Alpha was computed for the FORS (α = 0.61) and IIRS (α = 0.69) measures to assess internal consistency; both were moderately reliable. Differences between the pre- and post-composite scores for the FORS and IIRS survey were compared using Kruskal–Wallis tests. *P* values less than 0.05 were considered statistically significant. All analyses were performed using SAS (SAS Institute, version 9.4).

## Results

20 clinicians participated in the study.

### Clinician participant demographic characteristics

Of the 20 clinicians who participated in the study, three were consultant physicians, eight were Advanced Practice Providers (including nurse practitioners and physician assistants), and nine were multidisciplinary Fellows in Pulmonary/Critical Care and Hematology/Oncology. Seventy percent (*n* =14) of the clinicians were female, and 50% (*n* = 10) reported being born outside of the United States. Forty percent (*n* = 8) of the participating clinicians were White, with 40% (*n* = 8) identifying as Asian/South Asian or Asian Pacific. Full demographic information is reported in Table 1.

**Table 1.** Demographics

	Control (*N* = 9)	Intervention (*N* = 11)	Total (*N* = 20)
Role, *n* (%)			
Attending physician	1 (11.1%)	2 (18.2%)	3 (15.0%)
Advanced practice provider (nurse practitioner, physician assistant)	4 (44.4%)	4 (36.4%)	8 (40.0%)
Resident or fellow physician	4 (44.4%)	5 (45.5%)	9 (45.0%)
How many years of post-graduate work experience do you have, approximately (including residency and fellowship)?, *n* (%)			
Less than 5	2 (22.2%)	2 (18.2%)	4 (20.0%)
5–10	3 (33.3%)	8 (72.7%)	11 (55.0%)
11–15	4 (44.4%)	1 (9.1%)	5 (25.0%)
For Physician staff, what is your primary board certification?, *n* (%)			
Internal medicine	5 (100.0%)	5 (83.3%)	10 (90.9%)
Other	0 (0.0%)	1 (16.7%)	1 (9.1%)
Resident/Fellow	4	5	9
For Residents/Fellows, what is your training program?, *n* (%)			
Pulmonary/Critical care	1 (33.3%)	2 (33.3%)	3 (33.3%)
Hematology/Oncology	1 (33.3%)	2 (33.3%)	3 (33.3%)
Other	1 (33.3%)	2 (33.3%)	3 (33.3%)
MD staff	6	5	11
Gender, *n* (%)			
Female	7 (77.8%)	7 (63.6%)	14 (70.0%)
Male	2 (22.2%)	4 (36.4%)	6 (30.0%)
Race and ethnicity (check all that apply), *n* (%)			
Asian, South Asian, or Asian Pacific	4 (44.4%)	4 (36.4%)	8 (40.0%)
Black, African, or African American	1 (11.1%)	0 (0.0%)	1 (5.0%)
Middle Eastern	0 (0.0%)	1 (9.1%)	1 (5.0%)
White	3 (33.3%)	5 (45.5%)	8 (40.0%)
Other	1 (11.1%)	1 (9.1%)	2 (10.0%)
Were you born in the USA?, *n* (%)			
No	5 (55.6%)	5 (45.5%)	10 (50.0%)
Yes	4 (44.4%)	6 (54.5%)	10 (50.0%)

Table 2 presents the results of the pre-session survey. While the majority (80%) of participants (*n* =16) worked with interpreters once or less per week, 80% (*n* = 16) felt confident working with interpreters, and 44.5% (*n* = 8) reported receiving some training in interpreter-mediated communication at some point.

**Table 2.** Pre-Session Survey

	Control (*N* = 9)	Intervention (*N* = 11)	Total (*N* = 20)
I often care for patients with a serious illness and language barriers, *n* (%)			
Less than once a week	2 (22.2%)	5 (45.5%)	7 (35.0%)
About once a week	4 (44.4%)	2 (18.2%)	6 (30.0%)
More than once a week	1 (11.1%)	1 (9.1%)	2 (10.0%)
Most days	2 (22.2%)	3 (27.3%)	5 (25.0%)
I often work with medical interpreters, *n* (%)			
Less than once a week	3 (33.3%)	6 (54.5%)	9 (45.0%)
About once a week	5 (55.6%)	2 (18.2%)	7 (35.0%)
More than once a week	1 (11.1%)	2 (18.2%)	3 (15.0%)
Most days	0 (0.0%)	1 (9.1%)	1 (5.0%)
I am comfortable conducting a medical encounter in a language other than English, *n* (%)			
Strongly disagree	1 (11.1%)	2 (18.2%)	3 (15.0%)
Disagree	2 (22.2%)	3 (27.3%)	5 (25.0%)
Neither agree nor disagree	2 (22.2%)	3 (27.3%)	5 (25.0%)
Agree	3 (33.3%)	2 (18.2%)	5 (25.0%)
Strongly agree	1 (11.1%)	1 (9.1%)	2 (10.0%)
I have received some training in interpreter-mediated communication, *n* (%)			
Strongly disagree	2 (25.0%)	2 (20.0%)	4 (22.2%)
Disagree	1 (12.5%)	2 (20.0%)	3 (16.7%)
Neither agree nor disagree	2 (25.0%)	1 (10.0%)	3 (16.7%)
Agree	3 (37.5%)	4 (40.0%)	7 (38.9%)
Strongly agree	0 (0.0%)	1 (10.0%)	1 (5.6%)
Missing	1	1	2
I feel confident working with medical interpreters, *n* (%)			
Neither agree nor disagree	1 (11.1%)	3 (27.3%)	4 (20.0%)
Agree	8 (88.9%)	6 (54.5%)	14 (70.0%)
Strongly agree	0 (0.0%)	2 (18.2%)	2 (10.0%)

Table 3 presents the post-session survey completed by all participants. Although 90% (*n* = 18) reported that their previous medical training had prepared them for such an encounter, 94.8% (*n* = 18) indicated that the session increased their confidence in working with interpreters. All participants (*n* = 20) felt that the scenario was realistic.

**Table 3.** Post-Session Survey

	Control (*N* = 9)	Intervention (*N* = 11)	Total (*N* = 20)
My previous medical education training prepared me for this session, *n* (%)			
Disagree	1 (11.1%)	1 (9.1%)	2 (10.0%)
Agree	7 (77.8%)	8 (72.7%)	15 (75.0%)
Strongly agree	1 (11.1%)	2 (18.2%)	3 (15.0%)
This session increased my confidence with working with interpreters, *n* (%)			
Neither agree nor disagree	0 (0.0%)	1 (9.1%)	1 (5.3%)
Agree	6 (75.0%)	6 (54.5%)	12 (63.2%)
Strongly agree	2 (25.0%)	4 (36.4%)	6 (31.6%)
Missing	1	0	1
The simulation scenario felt realistic, *n* (%)			
Agree	5 (55.6%)	5 (45.5%)	10 (50.0%)
Strongly agree	4 (44.4%)	6 (54.5%)	10 (50.0%)

The participants who used the guide had favorable opinions of it. Table 4 presents the feasibility survey results completed by the intervention group (*n* = 11). Ninety-one percent of participants (*n* = 10) agreed/strongly agreed with the acceptability of the tool, 91% (*n* =10) agreed/strongly agreed with the appropriateness of the tool, and all (*n* =11) agreed/strongly agreed with the feasibility of the tool.

**Table 4.** Post Session CHECK-IN Guide Feasibility Questions

	Intervention (*N* = 11)
The CHECK-IN guide is appealing to me, *n* (%)	
Neither agree or disagree	1 (9.1%)
Agree	5 (45.5%)
Strongly agree	5 (45.5%)
I like the CHECK-IN guide, *n* (%)	
Neither agree or disagree	1 (9.1%)
Agree	5 (45.5%)
Strongly agree	5 (45.5%)
The CHECK-IN guide seems suitable for clinical practice, *n* (%)	
Neither agree nor disagree	1 (9.1%)
Agree	5 (45.5%)
Strongly agree	5 (45.5%)
The CHECK-IN guide seems applicable for clinical practice, *n* (%)	
Neither agree nor disagree	1 (9.1%)
Agree	5 (45.5%)
Strongly agree	5 (45.5%)
The CHECK-IN guide seems doable in clinical practice, *n* (%)	
Agree	4 (36.4%)
Strongly agree	7 (63.6%)
The CHECK-IN guide seems easy to use, *n* (%)	
Agree	5 (45.5%)
Strongly agree	6 (54.5%)
The CHECK-IN guide would help me communicate more effectively with culturally and linguistically diverse patients, *n* (%)	
Neither agree nor disagree	1 (9.1%)
Agree	4 (36.4%)
Strongly agree	6 (54.5%)

CHECK-IN, Check in for Exchange of Clinical and Key Information.

The IS, IIRS, and FORS scores were similar between the control and interventions groups. See Supplementary Tables S5–S7 are in Supplementary Appendix A3. For the checklist, answer categories were combined due to the small sample size, with ‘Poor’ and ‘Fair’ being combined, and ‘Good’, ‘Very Good’, and ‘Excellent’ being combined. Only one domain “potential challenges were discussed” was statistically significantly different between the control and intervention groups. See Supplementary Table S8 in Supplementary Appendix A3.

We also invited participants to provide feedback on the CHECK-IN guide.

Below are some of the participant comments:

*“The premise of the study is important. Having these conversations without language barriers is difficult; with language barriers, it gets harder. Usually there is no huddle or interpreter and physician conversation before communicating with the patient, but this made me appreciate its importance.”*

*“I think the CHECK-IN guide is incredibly helpful and the huddle between the clinician and interpreter is important to have a clear plan for the encounter.”*

*“I think using it (CHECK-IN guide) as a starting point is an excellent reminder to facilitate conversation with the interpreter before diving in. I’ve never considered explaining the clinical scenario to the interpreter before entering the patient room but plan to do this in the future because I do feel it leads to improved patient care. Giving difficult news is never easy but becomes less challenging when anticipated and discussed ahead of time.”*

*“CHECK-IN guide caused me to listen more… .seems very helpful to make sure interpreters get to contribute at their full level.”*

*“I don’t think the CHECK-IN guide needs to be used in a systematic manner to be useful. I think using it as a starting point is an excellent reminder to facilitate conversation with the interpreter before diving in.”*

## Discussion

This pilot study aimed to evaluate the feasibility and preliminary performance of a simple communication tool, the CHECK-IN guide among clinicians. While best practice guidelines recommend holding a pre-encounter huddle between clinician and interpreter, there is little evidence to guide the content of this huddle or measure the impact of the pre-encounter huddle on the quality of communication during the patient encounter/family meeting.

Our study found that the CHECK-IN guide was viewed favorably by the clinician participants, and scored highly on acceptability, appropriateness, and feasibility scores. The study did not detect any statistically significant differences in communication performance between the intervention group and the control group, with participants receiving similar scores from the study’s interpreter, SPs, and faculty. This could be in part due to the small sample size, but also the result of participants’ previous training and interactions with interpreters.

Existing previous research has established that patients with non-English language preference have worse health outcomes in a variety of settings, including within hospice and palliative care.^2–4,6,7^ Thus, it is critical to systemically address barriers faced by these patients with non-English language preference to ensure the highest quality care for this vulnerable population and develop interventions to address them. While professional medical interpretation is not always a panacea due to lack of access or inconsistent training/certification, effective collaboration with medical interpreters has been shown to improve the quality of care and patient satisfaction.^8,9,24,25^ A pre-encounter huddle is one step that could aid in improving collaboration.

Our study shows that the CHECK-IN guide is easy to use and acceptable to clinicians. It was helpful in providing a structure for the pre-encounter huddle. The written feedback from participating clinicians was particularly impactful. For some, having a pre-encounter huddle was a totally new concept. Many felt that the guide was very helpful in prompting clinicians to have a huddle with the interpreter and that the pre-encounter huddle help with formulating a plan for the patient encounter. It also provided a good opportunity to discuss and brainstorm how to approach potential cultural and linguistic barriers.

### Strengths

To our knowledge, this is the first study of its kind to evaluate a convenient bedside communication guide designed to facilitate bi-directional information and cultural exchange between a clinician and interpreter before a clinical encounter with a patient experiencing serious illness. Our study team included palliative care physicians, a chaplain, a Spanish interpreter, communications researcher, and health disparities researcher. Our study was developed in collaboration with medical interpreters, standardized patients, and simulation center staff. We used previously validated rating scales for faculty, interpreters, and standardized patients to evaluate encounters.

### Limitations

There are several limitations to our study.

Despite intensive efforts and escalating incentives, our study did not meet our initial recruitment target, which may have limited our ability to determine statistical differences between the intervention and control groups. The demographics of our study population suggest a degree of selection bias that may limit generalizability of our findings. For instance, 80% of our participants indicated that they felt prepared for a session involving interpreter-facilitated communication based on their medical education, while previous research suggests that only 20–50% of graduating medical students feel prepared for such an encounter^26,27^ Non-white participants and females were both over-represented in our study population as compared to our institutional demographics; 60% of our participants identified as a race other than white (vs. 23.4% of the clinicians at our institution overall) and 70% of our participants identified as female (vs. 51.0% of the clinicians at our institution overall). Furthermore, 50% of the participants were born outside of the United States and their own experiences may have impacted their attitudes and experiences with medical interpreters. In addition, it is possible that those who feel comfortable conducting these conversations and working with interpreters were more likely to enroll in the study, causing selection bias.

Quality of communication in serious illness conversations is also difficult to measure. Although we used validated scales for the patient encounter, the IS, IIRS. and FORS are behavior-anchored scales for interpreter-mediated encounters, but not specific to serious illness conversations. In addition, the IS includes some measures that are traditionally viewed as the interpreters’ responsibility rather than the clinicians’, e.g., introducing the interpreter. We added a checklist to observe cultural considerations that would be relevant during serious illness conversations and EOL cares. Future studies in the clinical setting might consider evaluating patient and family palliative quality metrics of feeling heard and feeling understood.

We only assessed clinicians in this study. It will be important to expand the research to evaluate the tool with interpreters as research participants as well. Additionally, medical interpretation currently includes remote interpretation including phone and video interpreters, in addition to in-person interpreters. Our study only focused on in-person interpreters and future research should include remote modalities of interpretation and whether CHECK-IN is feasible in those encounters.

Lastly, our study was also conducted in a controlled simulation-based setting and while the participants agreed that the scenario was realistic, this is not the same as a clinical setting.

## Conclusion

Patients with non-English language preference are increasingly likely to engage in serious illness conversations. Successful collaboration with professional medical interpreters is an essential part of a comprehensive process for mitigating established health care disparities for this underserved population. Although previous research has suggested that a pre-encounter huddle is a valuable component of an interpreter-facilitated medical encounter, there is very little evidence to guide the content of this huddle. Our CHECK-IN tool was developed to facilitate efficient and high-quality communication between a clinician and interpreter before a visit with a patient experiencing serious illness. Our results demonstrate the feasibility and acceptability of the tool in a simulated patient encounter. Further research should evaluate the tool across the spectrum of medical education and across different clinical settings and languages to better characterize its value and, if needed, help to iteratively refine the tool.

## Notes

Supplementary Appendix A1

Supplementary Appendix A2

Supplementary Appendix A3

## Abbreviations Used

CHECK-IN: Check in for Exchange of Clinical and Key Information
EOL: end of life
FORS: Faculty Observation Rating Scale
IIRS: Interpreter Impact Rating Scale
IS: Interpreter Scale
SP: Standardized Patient
