# Supplementary material for: Feasibility, Acceptability, and Preliminary Performance of Check In for Exchange of Clinical and Key Information: A Communication Guide to Facilitate Pre-Encounter Huddles with Medical Interpreters Prior to Conversations Around Serious Illness
Source: Palliat Med Rep. 2025 Oct 27;6(1):533–41. doi: 10.1177/26892820251390817 (PMC12670661; doi:10.1177/26892820251390817)
Supplement: Supplementary Appendix A1 [file 26892820251390817_supplementary_appendix_a1.docx]

Appendix 1: Case Scenario Information for Study Participant

Mr C is an 76 year old patient with metastatic colon cancer. He is admitted to the hospital for management of cancer pain; imaging shows new peritoneal carcinomatosis and progression of liver metastases despite going through multiple lines of treatments. Oncology is consulted and is recommending no further cancer directed treatments. Prognosis is estimated at weeks to a few months. His symptoms are now controlled on a complex pain regimen. Her PCP and oncologist are not comfortable managing this pain regimen and recommend that patient enroll with home hospice after discharge for symptom management and end of life care.

You are the representative clinician from the primary inpatient service and have arranged a care conference to discuss goals of care and disposition. Mr C speaks fluent Spanish and does not speak any English. Patient’s family member is also present and is bilingual and has been interpreting for the patient. As this is a high stakes conversation, you have arranged for a medical interpreter to be present for this encounter.

You meet with the interpreter in room A. If you have received a copy of the CHECK-IN guide, feel free to use the guide with the interpreter

When you and the interpreter are ready, both of you will enter the simulation room.

Other pertinent information:

Mr C immigrated from Mexico to the US in her/his 40’s.

Patient is widowed, with 3 children and 5 grandchildren

Patient’s family member is his/her main caregiver and has been to all the medical appointments
