# Supplementary material for: Feasibility, Acceptability, and Preliminary Performance of Check In for Exchange of Clinical and Key Information: A Communication Guide to Facilitate Pre-Encounter Huddles with Medical Interpreters Prior to Conversations Around Serious Illness
Source: Palliat Med Rep. 2025 Oct 27;6(1):533–41. doi: 10.1177/26892820251390817 (PMC12670661; doi:10.1177/26892820251390817)
Supplement: Supplementary Appendix A2 [file 26892820251390817_supplementary_appendix_a2.docx]

Appendix 2: IS, IIRS, FORS, Checklist

Interpreter Scale (IS)

To be completed by the Interpreter

|  | 1  Marginal/Low  Performance | 2 | 3 | 4 | 5  Outstanding  Performance |
| --- | --- | --- | --- | --- | --- |
| The participant introduced himself/herself to me |  |  |  |  |  |
| The participant introduced me to the patient |  |  |  |  |  |
| The participant adequately explained the purpose of the interview |  |  |  |  |  |
| The participant explained my role to the patient at the beginning |  |  |  |  |  |
| The participant arrange the seating in a manner conducive to effective interpretation |  |  |  |  |  |
| The participant asked the patient one question at a time |  |  |  |  |  |
| The participant listened to me as I interpreted the patient’s answers, without unnecessary interruptions |  |  |  |  |  |
| The participant asked questions to clarify his/her own understanding of the patient’s answers |  |  |  |  |  |
| The participant asked the patient if he/she had any questions |  |  |  |  |  |
| The participant maintained direct eye contact with the patient (instead of me) most of the time |  |  |  |  |  |
| The participant addressed the patient in the first person and not as “he/she” |  |  |  |  |  |
| The participant kept me ‘on track’ (ie questioned me when lapses led to incomplete interpretations |  |  |  |  |  |
| Rate your overall satisfaction with the encounter |  |  |  |  |  |

Interpreter Impact Rating Scale (IIRS)

To be completed by the Actor/Standardized Patient

|  | 1  Marginal/low  performance | 2 | 3 | 4 | 5  Outstanding  Performance |
| --- | --- | --- | --- | --- | --- |
| Participant showed direct eye contact with me during the encounter instead of at the interpreter most of the time. |  |  |  |  |  |
| Participant directly addressed the issues translated that were of concern to me. |  |  |  |  |  |
| Participant acknowledged and responded to my beliefs, concerns, and expectations about my problems. |  |  |  |  |  |
| Participant asked me questions in the first person (example: “Do you feel...” rather than “interpreter, can you ask him if he...”) |  |  |  |  |  |
| Participant sat at a comfortable distance from me (not too close and not too far away). |  |  |  |  |  |
| Participant’s nonverbal body communication was reassuring (i.e.,—mannerisms, facial expressions, and body language). |  |  |  |  |  |
| Rate your overall satisfaction with the encounter. |  |  |  |  |  |

Faculty Observer Rating Scale (FORS)

To be completed by Faculty

|  | 1  Marginal/Low  Performance | 2 | 3 | 4 | 5  Outstanding Performance |
| --- | --- | --- | --- | --- | --- |
| The participant adequately explained the purpose of the interview to the interpreter. |  |  |  |  |  |
| The participant explained the interpreter’s role to the patient at the beginning. |  |  |  |  |  |
| The participant asked the patient one question at a time. |  |  |  |  |  |
| The participant listened to the patient without unnecessary interruption. |  |  |  |  |  |
| The participant asked questions to clarify his/her own understanding of the patient’s answers |  |  |  |  |  |
| The participant presented information at a pace that was easy to follow for both patient and interpreter; that is, information was given in “digestible chunks”. |  |  |  |  |  |
| The participant maintained direct eye contact with the patient (instead of the interpreter). |  |  |  |  |  |

CHECK-LIST (to be filled out by faculty)

Pre-Encounter Huddle

On a scale of 1-5 with 1 being poor and 5 being excellent, how well did the participant:

|  | 1 | 2 | 3 | 4 | 5 |
| --- | --- | --- | --- | --- | --- |
| Explain the purpose of the meeting |  |  |  |  |  |
| Provide medical background to the interpreter |  |  |  |  |  |
| Ask about cultural/linguistic aspects of care |  |  |  |  |  |
| Discuss potential challenges |  |  |  |  |  |
| Clarify roles |  |  |  |  |  |

Patient Encounter

On a scale of 1-5 1 being poor and 5 being excellent, how well did the participant:

|  | 1 | 2 | 3 | 4 | 5 |
| --- | --- | --- | --- | --- | --- |
| Ask about patient preferences for receiving information and decision-making |  |  |  |  |  |
| Use teach-back technique to ensure understanding |  |  |  |  |  |
| Explore the role of culture and religion when discussing goals of care |  |  |  |  |  |
| Explains hospice in a culturally sensitive manner |  |  |  |  |  |
