# Supplementary material for: Feasibility, Acceptability, and Preliminary Performance of Check In for Exchange of Clinical and Key Information: A Communication Guide to Facilitate Pre-Encounter Huddles with Medical Interpreters Prior to Conversations Around Serious Illness
Source: Palliat Med Rep. 2025 Oct 27;6(1):533–41. doi: 10.1177/26892820251390817 (PMC12670661; doi:10.1177/26892820251390817)
Supplement: Supplementary Appendix A3 [file 26892820251390817_supplementary_appendix_a3.docx]

Appendix/supplemental materials 3: Tables 5-8

Table 5: IS Scale (completed by the interpreter)

|  | Control (N=9) | Intervention (N=11) | Total (N=20) |
| --- | --- | --- | --- |
| The participant introduced himself/herself to me, n (%) |  |  |  |
| 3 | 1 (11.1%) | 1 (9.1%) | 2 (10.0%) |
| 4 | 3 (33.3%) | 2 (18.2%) | 5 (25.0%) |
| 5 | 5 (55.6%) | 8 (72.7%) | 13 (65.0%) |
|  |  |  |  |
| The participant introduced me to the patient, n (%) |  |  |  |
| 1 | 1 (12.5%) | 3 (30.0%) | 4 (22.2%) |
| 2 | 0 (0.0%) | 1 (10.0%) | 1 (5.6%) |
| 3 | 0 (0.0%) | 1 (10.0%) | 1 (5.6%) |
| 4 | 2 (25.0%) | 1 (10.0%) | 3 (16.7%) |
| 5 | 5 (62.5%) | 4 (40.0%) | 9 (50.0%) |
| Missing | 1 | 1 | 2 |
|  |  |  |  |
| The participant adequately explained the purpose of the interview, n (%) |  |  |  |
| 3 | 1 (11.1%) | 2 (18.2%) | 3 (15.0%) |
| 4 | 0 (0.0%) | 2 (18.2%) | 2 (10.0%) |
| 5 | 8 (88.9%) | 7 (63.6%) | 15 (75.0%) |
|  |  |  |  |
| The participant explained my role to the patient at the beginning, n (%) |  |  |  |
| 1 | 1 (11.1%) | 5 (50.0%) | 6 (31.6%) |
| 2 | 1 (11.1%) | 0 (0.0%) | 1 (5.3%) |
| 3 | 0 (0.0%) | 1 (10.0%) | 1 (5.3%) |
| 4 | 3 (33.3%) | 0 (0.0%) | 3 (15.8%) |
| 5 | 4 (44.4%) | 4 (40.0%) | 8 (42.1%) |
| Missing | 0 | 1 | 1 |
|  |  |  |  |
| The participant arrange the seating in a manner conducive to effective interpretation, n (%) |  |  |  |
| 1 | 1 (11.1%) | 0 (0.0%) | 1 (5.0%) |
| 2 | 0 (0.0%) | 1 (9.1%) | 1 (5.0%) |
| 3 | 2 (22.2%) | 2 (18.2%) | 4 (20.0%) |
| 4 | 2 (22.2%) | 2 (18.2%) | 4 (20.0%) |
| 5 | 4 (44.4%) | 6 (54.5%) | 10 (50.0%) |
|  |  |  |  |
| The participant asked the patient one question at a time, n (%) |  |  |  |
| 3 | 3 (33.3%) | 3 (30.0%) | 6 (31.6%) |
| 4 | 1 (11.1%) | 1 (10.0%) | 2 (10.5%) |
| 5 | 5 (55.6%) | 6 (60.0%) | 11 (57.9%) |
| Missing | 0 | 1 | 1 |
|  |  |  |  |
| The participant listened to me as I interpreted the patient's answers, without unnecessary interruptions, n (%) |  |  |  |
| 3 | 0 (0.0%) | 1 (9.1%) | 1 (5.0%) |
| 5 | 9 (100.0%) | 10 (90.9%) | 19 (95.0%) |
|  |  |  |  |
| The participant asked questions to clarify his/her own understanding of the patient's answers, n (%) |  |  |  |
| 2 | 1 (14.3%) | 1 (12.5%) | 2 (13.3%) |
| 3 | 1 (14.3%) | 1 (12.5%) | 2 (13.3%) |
| 4 | 3 (42.9%) | 4 (50.0%) | 7 (46.7%) |
| 5 | 2 (28.6%) | 2 (25.0%) | 4 (26.7%) |
| Missing | 2 | 3 | 5 |
|  |  |  |  |
| The participant asked the patient is he/she had any questions, n (%) |  |  |  |
| 2 | 1 (11.1%) | 0 (0.0%) | 1 (5.0%) |
| 3 | 1 (11.1%) | 2 (18.2%) | 3 (15.0%) |
| 4 | 1 (11.1%) | 1 (9.1%) | 2 (10.0%) |
| 5 | 6 (66.7%) | 8 (72.7%) | 14 (70.0%) |
|  |  |  |  |
| The participant maintained direct eye contact with the patient (instead of me) most of the time, n (%) |  |  |  |
| 2 | 0 (0.0%) | 1 (9.1%) | 1 (5.3%) |
| 3 | 1 (12.5%) | 1 (9.1%) | 2 (10.5%) |
| 5 | 7 (87.5%) | 9 (81.8%) | 16 (84.2%) |
| Missing | 1 | 0 | 1 |
|  |  |  |  |
| The participant addressed the patient in the first person and not as he/she, n (%) |  |  |  |
| 2 | 0 (0.0%) | 1 (9.1%) | 1 (5.3%) |
| 3 | 1 (12.5%) | 0 (0.0%) | 1 (5.3%) |
| 4 | 1 (12.5%) | 1 (9.1%) | 2 (10.5%) |
| 5 | 6 (75.0%) | 9 (81.8%) | 15 (78.9%) |
| Missing | 1 | 0 | 1 |
|  |  |  |  |
| The participant kept me 'on track' (ie questioned me when lapses led to incomplete interpretations, n (%) |  |  |  |
| 1 | 0 (0.0%) | 1 (16.7%) | 1 (9.1%) |
| 3 | 1 (20.0%) | 1 (16.7%) | 2 (18.2%) |
| 4 | 1 (20.0%) | 3 (50.0%) | 4 (36.4%) |
| 5 | 3 (60.0%) | 1 (16.7%) | 4 (36.4%) |
| Missing | 4 | 5 | 9 |
|  |  |  |  |
| Rate your overall satisfaction with the encounter, n (%) |  |  |  |
| 3 | 0 (0.0%) | 1 (9.1%) | 1 (5.0%) |
| 4 | 4 (44.4%) | 5 (45.5%) | 9 (45.0%) |
| 5 | 5 (55.6%) | 5 (45.5%) | 10 (50.0%) |

Table 6: individual questions for IIRS (completed by the 2 actors)

|  | Control (N=18) | Intervention (N=22) | Total (N=40) |
| --- | --- | --- | --- |
| Participant showed direct eye contact with me during the encounter instead of at the interpreter most of the time., n (%) |  |  |  |
| 4 | 2 (11.1%) | 6 (27.3%) | 8 (20.0%) |
| 5 – Outstanding Performance | 16 (88.9%) | 16 (72.7%) | 32 (80.0%) |
|  |  |  |  |
| Participant directly addressed the issues translated that were of concern to me., n (%) |  |  |  |
| 3 | 1 (5.6%) | 3 (13.6%) | 4 (10.0%) |
| 4 | 4 (22.2%) | 9 (40.9%) | 13 (32.5%) |
| 5 – Outstanding Performance | 13 (72.2%) | 10 (45.5%) | 23 (57.5%) |
|  |  |  |  |
| Participant acknowledged and responded to my beliefs, concerns, and expectations about my problems., n (%) |  |  |  |
| 2 | 0 (0.0%) | 1 (4.5%) | 1 (2.5%) |
| 3 | 2 (11.1%) | 1 (4.5%) | 3 (7.5%) |
| 4 | 5 (27.8%) | 12 (54.5%) | 17 (42.5%) |
| 5 – Outstanding Performance | 11 (61.1%) | 8 (36.4%) | 19 (47.5%) |
|  |  |  |  |
| Participant asked me questions in the first person (example: Do you feel... rather than interpreter, can you ask him if he...), n (%) |  |  |  |
| 1 – Marginal/Low Performance | 3 (16.7%) | 0 (0.0%) | 3 (7.5%) |
| 3 | 1 (5.6%) | 1 (4.5%) | 2 (5.0%) |
| 4 | 1 (5.6%) | 5 (22.7%) | 6 (15.0%) |
| 5 – Outstanding Performance | 13 (72.2%) | 16 (72.7%) | 29 (72.5%) |
|  |  |  |  |
| Participant sat at a comfortable distance from me (not too close and not too far away)., n (%) |  |  |  |
| 1 – Marginal/Low Performance | 0 (0.0%) | 1 (4.5%) | 1 (2.5%) |
| 3 | 3 (16.7%) | 3 (13.6%) | 6 (15.0%) |
| 4 | 5 (27.8%) | 11 (50.0%) | 16 (40.0%) |
| 5 – Outstanding Performance | 10 (55.6%) | 7 (31.8%) | 17 (42.5%) |
|  |  |  |  |
| Participant's nonverbal body communication was reassuring (i.e.,-mannerisms, facial expressions, and body language)., n (%) |  |  |  |
| 2 | 1 (5.6%) | 2 (9.1%) | 3 (7.5%) |
| 3 | 2 (11.1%) | 4 (18.2%) | 6 (15.0%) |
| 4 | 8 (44.4%) | 7 (31.8%) | 15 (37.5%) |
| 5 – Outstanding Performance | 7 (38.9%) | 9 (40.9%) | 16 (40.0%) |
|  |  |  |  |
| Rate your overall satisfaction with the encounter., n (%) |  |  |  |
| 3 | 2 (11.1%) | 3 (13.6%) | 5 (12.5%) |
| 4 | 7 (38.9%) | 10 (45.5%) | 17 (42.5%) |
| 5 – Outstanding Performance | 9 (50.0%) | 9 (40.9%) | 18 (45.0%) |

*Answers went from 1 to 5, with 1 being Marginal/Low Performance and 5 being Outstanding Performance

Table 7 individual questions for FORS (completed by 2 observing faculty)

|  | Control (N=18) | Intervention (N=22) | Total (N=40) |
| --- | --- | --- | --- |
| The participant adequately explained the purpose of the interview to the interpreter., n (%) |  |  |  |
| 1 – Marginal/Low Performance | 0 (0.0%) | 3 (14.3%) | 3 (7.7%) |
| 2 | 1 (5.6%) | 1 (4.8%) | 2 (5.1%) |
| 3 | 1 (5.6%) | 1 (4.8%) | 2 (5.1%) |
| 4 | 5 (27.8%) | 6 (28.6%) | 11 (28.2%) |
| 5 – Outstanding Performance | 11 (61.1%) | 10 (47.6%) | 21 (53.8%) |
| Missing | 0 | 1 | 1 |
|  |  |  |  |
| The participant explained the interpreter's role to the patient at the beginning., n (%) |  |  |  |
| 1 – Marginal/Low Performance | 3 (16.7%) | 9 (45.0%) | 12 (31.6%) |
| 2 | 3 (16.7%) | 3 (15.0%) | 6 (15.8%) |
| 3 | 5 (27.8%) | 3 (15.0%) | 8 (21.1%) |
| 4 | 2 (11.1%) | 1 (5.0%) | 3 (7.9%) |
| 5 – Outstanding Performance | 5 (27.8%) | 4 (20.0%) | 9 (23.7%) |
| Missing | 0 | 2 | 2 |
|  |  |  |  |
| The participant asked the patient one question at a time., n (%) |  |  |  |
| 3 | 0 (0.0%) | 3 (14.3%) | 3 (7.7%) |
| 4 | 4 (22.2%) | 5 (23.8%) | 9 (23.1%) |
| 5 – Outstanding Performance | 14 (77.8%) | 13 (61.9%) | 27 (69.2%) |
| Missing | 0 | 1 | 1 |
|  |  |  |  |
| The participant listened to the patient without unnecessary interruption., n (%) |  |  |  |
| 4 | 2 (11.1%) | 7 (31.8%) | 9 (22.5%) |
| 5 – Outstanding Performance | 16 (88.9%) | 15 (68.2%) | 31 (77.5%) |
|  |  |  |  |
| The participant asked questions to clarify his/her own understanding of the patient's answers, n (%) |  |  |  |
| 1 – Marginal/Low Performance | 2 (11.1%) | 1 (4.5%) | 3 (7.5%) |
| 2 | 4 (22.2%) | 2 (9.1%) | 6 (15.0%) |
| 3 | 8 (44.4%) | 7 (31.8%) | 15 (37.5%) |
| 4 | 3 (16.7%) | 5 (22.7%) | 8 (20.0%) |
| 5 – Outstanding Performance | 1 (5.6%) | 7 (31.8%) | 8 (20.0%) |
|  |  |  |  |
| The participant presented information at a pace that was easy to follow for both patient and interpreter; that is, information was given in digestible chunks., n (%) |  |  |  |
| 2 | 1 (5.9%) | 0 (0.0%) | 1 (2.6%) |
| 3 | 2 (11.8%) | 1 (4.5%) | 3 (7.7%) |
| 4 | 7 (41.2%) | 7 (31.8%) | 14 (35.9%) |
| 5 – Outstanding Performance | 7 (41.2%) | 14 (63.6%) | 21 (53.8%) |
| Missing | 1 | 0 | 1 |
|  |  |  |  |
| The participant maintained direct eye contact with the patient (instead of the interpreter)., n (%) |  |  |  |
| 3 | 1 (5.6%) | 0 (0.0%) | 1 (2.5%) |
| 4 | 2 (11.1%) | 2 (9.1%) | 4 (10.0%) |
| 5 – Outstanding Performance | 15 (83.3%) | 20 (90.9%) | 35 (87.5%) |

*Answers went from 1 to 5, with 1 being Marginal/Low Performance and 5 being Outstanding Performance

Table 8: CheckList: Pre-Encounter checklist and Encounter Checklist (completed by 2 observing faculty)

|  | Control (N=18) | Intervention  (N=22) | Total (N=40) | P-value |
| --- | --- | --- | --- | --- |
| Explain the purpose of the meeting, n (%) |  |  |  | 0.1071^1^ |
| Poor/Fair | 0 (0.0%) | 4 (20.0%) | 4 (10.5%) |  |
| Good/Very Good/Excellent | 18 (100.0%) | 16 (80.0%) | 34 (89.5%) |  |
| Missing | 0 | 2 | 2 |  |
|  |  |  |  |  |
| Provide medical background to the interpreter, n (%) |  |  |  | 0.3436^1^ |
| Poor/Fair | 1 (5.6%) | 4 (20.0%) | 5 (13.2%) |  |
| Good/Very Good/Excellent | 17 (94.4%) | 16 (80.0%) | 33 (86.8%) |  |
| Missing | 0 | 2 | 2 |  |
|  |  |  |  |  |
| Ask about cultural/linguistic aspects of care, n (%) |  |  |  | 0.7342^1^ |
| Poor/Fair | 7 (38.9%) | 6 (30.0%) | 13 (34.2%) |  |
| Good/Very Good/Excellent | 11 (61.1%) | 14 (70.0%) | 25 (65.8%) |  |
| Missing | 0 | 2 | 2 |  |
|  |  |  |  |  |
| Discuss potential challenges, n (%) |  |  |  | 0.0029^1^ |
| Poor/Fair | 13 (76.5%) | 5 (25.0%) | 18 (48.6%) |  |
| Good/Very Good/Excellent | 4 (23.5%) | 15 (75.0%) | 19 (51.4%) |  |
| Missing | 1 | 2 | 3 |  |
|  |  |  |  |  |
| Clarify roles, n (%) |  |  |  | 1.0000^1^ |
| Poor/Fair | 13 (72.2%) | 13 (68.4%) | 26 (70.3%) |  |
| Good/Very Good/Excellent | 5 (27.8%) | 6 (31.6%) | 11 (29.7%) |  |
| Missing | 0 | 3 | 3 |  |
|  |  |  |  |  |
| Ask about patient preferences for receiving information and decision-making, n (%) |  |  |  | 0.4977^1^ |
| Poor/Fair | 6 (33.3%) | 5 (22.7%) | 11 (27.5%) |  |
| Good/Very Good/Excellent | 12 (66.7%) | 17 (77.3%) | 29 (72.5%) |  |
|  |  |  |  |  |
| Use teach-back technique to ensure understanding, n (%) |  |  |  | 1.0000^1^ |
| Poor/Fair | 16 (88.9%) | 19 (86.4%) | 35 (87.5%) |  |
| Good/Very Good/Excellent | 2 (11.1%) | 3 (13.6%) | 5 (12.5%) |  |
|  |  |  |  |  |
| Explore the role of culture and religion when discussing goals of care, n (%) |  |  |  | 0.1853^1^ |
| Poor/Fair | 9 (52.9%) | 6 (28.6%) | 15 (39.5%) |  |
| Good/Very Good/Excellent | 8 (47.1%) | 15 (71.4%) | 23 (60.5%) |  |
| Missing | 1 | 1 | 2 |  |
|  |  |  |  |  |
| Explains hospice in a culturally sensitive manner, n (%) |  |  |  | 0.2065^1^ |
| Poor/Fair | 2 (11.1%) | 0 (0.0%) | 2 (5.1%) |  |
| Good/Very Good/Excellent | 16 (88.9%) | 21 (100.0%) | 37 (94.9%) |  |
| Missing | 0 | 1 | 1 |  |

^1^Fisher Exact p-value;
